# Transoral thyroid and parathyroid surgery in Brazil: where are we?

**DOI:** 10.1590/0100-6991e-20233457-en

**Published:** 2023-05-04

**Authors:** LUCAS RIBEIRO TENÓRIO, ANTONIO AUGUSTO BERTELLI, MARIANNE YUMI NAKAI, MARCELO BENEDITO MENEZES, JONATHON OWEN RUSSELL, ANTONIO JOSÉ GONÇALVES

**Affiliations:** 1 - Faculdade de Ciências Médicas da Santa Casa de São Paulo, Disciplina de Cirurgia de Cabeça e Pescoço, Departamento de Cirurgia - São Paulo - SP - Brasil; 2 - Universidade Johns Hopkins, Divisão de Cirurgia Endócrina em Cabeça e Pescoço - Baltimore - MD - Estados Unidos

**Keywords:** TOETVA, Scarless Thyroid, Vestibular Approach, Thyroidectomy, Parathyroidectomy, Tireoidectomia, Paratireoidectomia, Neoplasias de Cabeça e Pescoço, Glândulas Endócrinas, Procedimentos Cirúrgicos Endócrinos

## Abstract

**Introduction:**

thyroid surgery through the transoral vestibular approach is a reality in many countries. While several competing remote access techniques have been developed in the last 20 years, many were not reproducible. Transoral Endoscopic Neck Surgery (TNS) has been shown to be reproducible in different centers around the world, and approximately five years after its description it has been adopted relatively quickly for various reasons. To date, there are at least 7 Brazilian studies published, including a series of more than 400 cases. The aim of this work is to study the progression of Transoral Neck Surgery in Brazil and describe the profile of surgeons involved in this new approach.

**Methods:**

this is a retrospective study with descriptive statistics. A REDCap based survey about transoral endoscopic thyroidectomy and parathyroidectomy vestibular approach (TOETVA/TOEPVA) was done with 66 Brazilian surgeons regarding surgeon profile, numbers of cases performed by geographic region, what kind of training was necessary prior to the first case and behavior of the surgeon proposing these new approaches.

**Results:**

response rate of this survey was 53%. To date, 1275 TOETVA/TOEPVA cases had been performed in Brazil, 1229 thyroidectomies (96.4%), 42 parathyroidectomies (3.3%) and 4 combined procedures (0.3%). Most of the cases were done in the southeast region (821, 64.4%), 538 (42.2%) cases in the State of São Paulo and 283 (22.2%) cases in the State of Rio de Janeiro.

**Conclusions:**

TOETVA is becoming popular in Brazil. Younger surgeons, especially those between 30 and 50 years old were more likely to adopt this approach.

## RESUMO

**Introdução:**

a cirurgia de tireoide por via transoral vestibular é uma realidade em muitos países. Embora várias outras técnicas de acesso remoto tenham sido desenvolvidas nos últimos 20 anos, muitas não eram reprodutíveis. A cirurgia endoscópica cervical transoral (TNS - Transoral Neck Surgery) tem se mostrado reprodutível em diferentes centros ao redor do mundo sendo que, aproximadamente cinco anos após sua descrição, foi adotada de forma relativamente rápida por vários motivos. Até o momento, existem pelo menos 7 estudos brasileiros publicados, incluindo uma série de mais de 400 casos. O objetivo deste trabalho é estudar a evolução da Cirurgia Transoral do Pescoço (TNS) no Brasil e descrever o perfil dos cirurgiões envolvidos nesta nova abordagem.

**Métodos:**

trata-se de um estudo descritivo. Uma pesquisa online hospedada no REDCap sobre tireoidectomia e paratireoidectomia endoscópica transoral por abordagem vestibular (TOETVA/TOEPVA) foi realizada com 66 cirurgiões brasileiros. Foram levantados dados sobre o perfil do cirurgião, número de casos realizados por região geográfica, que tipo de treinamento foi necessário antes do primeiro caso e comportamento do cirurgião durante o relacionamento com o paciente ao abordar essas novas técnicas.

**Resultados:**

a taxa de resposta desta pesquisa foi de 53%. Até o momento, 1.275 casos de TOETVA/TOEPVA foram realizados no Brasil, sendo 1.229 tireoidectomias (96,4%), 42 paratireoidectomias (3,3%) e 4 procedimentos combinados (0,3%). A maioria dos casos foi realizada na região sudeste (821, 64,4%), 538 (42,2%) casos no estado de São Paulo e 283 (22,2%) casos no estado do Rio de Janeiro.

**Conclusões:**

a TOETVA está se popularizando no Brasil. Cirurgiões mais jovens, especialmente aqueles entre 30 e 50 anos, são mais propensos a adotar essa abordagem.

## INTRODUCTION

**Figure 1:**
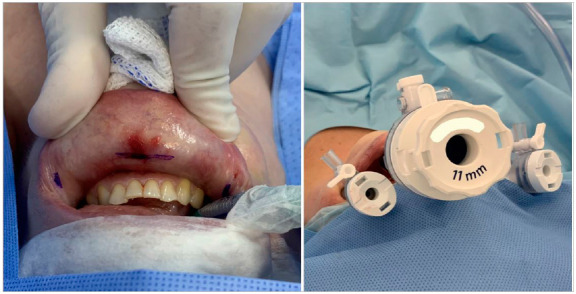
Image of the surgical access for TOETVA and TOEPVA.

Transoral thyroid and parathyroid endoscopic surgery through the vestibular approach is a reality in many countries in most parts of the world^1^ ^,^ ^2^. Several remote access thyroidectomy techniques have been developed in the last 20 years seeking for better cosmetic outcomes^3^ ^,^ ^4^. Many were not reproducible, and were therefore abandoned^5^. In Brazil, the first series of transoral endoscopic thyroidectomy was published in 2018 by Tesseroli et al.^6^. After that, more and more surgeons have adopted the technique and some have published their results^6^ ^-^ ^12^. This procedure is called Trans Oral Endoscopic Thyroidectomy/Parathyroidectomy Vestibular Approach (TOETVA/TOEPVA) by most authors to distinguish it from a previously described transoral technique that used one or more trocars through the floor of the mouth^13^. In this technique, surgical access is made with 3 trocars inserted through the oral vestibule (Figure 1). As such, the technique may be considered a natural orifice endoscopic surgery (NOTES™). Infection and other complications are rare, and the postoperative result is very similar to that of open thyroidectomy, but with the absence of a scar in the anterior neck^3^ ^,^ ^4^ ^,^ ^7^ ^,^ ^8^.

Other remote access thyroid surgery techniques, such as transaxillary, transthoracic and transmammary, are known to increase length of stay, require the systematic use of drains, increase recovery time and can result in serious complications not described in open thyroidectomy^5^. TOETVA demonstrates a safety profile that is similar to that of open surgery regarding complications related to the laryngeal nerves and parathyroid glands^4^. It allows access to both thyroid lobes and the central compartment. When compared to other remote access techniques, TOETVA is a true scarless surgery, as there are no cutaneous incisions. Of the remote access surgeries, it also represents the shortest route between the entry of the trocars and the thyroid, it doesn’t require routine drainage and brings a short length of stay and fast recovery. There is some evidence that there may be less pain with this technique as well^14^ ^-^ ^16^. Therefore TOETVA is considered a minimally invasive approach by some authors, although it may be best referred to as a remote access surgical technique^17^.

 TOETVA was first described by American surgeons^18^ after some experimental studies on animals and cadavers^13^ ^,^ ^18^ ^,^ ^19^, but it gained popularity after Anuwong, a surgeon from Thailand, published his first series of 60 cases in 2015, describing encouraging results^4^. The technique has been shown to be reproducible in different centers around the world, and has been adopted quickly^20^, as it requires only regular laparoscopic instruments without adding excessive costs and prohibitive surgical time. Further, it has been demonstrated to have a relatively short learning curve^8^ ^,^ ^9^. TOETVA also adds the benefits of videosurgery to thyroidectomy, such as image magnification and the ability to effortlessly record data and technique^3^ ^,^ ^21^. These video benefits may increase exponentially as artificial intelligence offers potential for surgical landmark assistance in the future. Cosmetically, it has been found to be superior when compared to open thyroidectomy^22^ ^,^ ^23^. 

To date, there are at least 7 Brazilian studies published about TOETVA^6^ ^-^ ^12^ including a series of more than 400 cases^7^. Other authors have described a brisk adoption of this technique relative to other remote access thyroidectomies^24^.

 Although TOETVA/TOEPVA has been adopted in other Latin American countries such as Argentina^25^ there is no evidence of the number of patients who have undergone this procedure in Brazil, nor the number of surgeons performing it. There are also some important concerns about the extent of surgical training surgeons are obtaining before starting TOETVA. 

The aim of this work is to study the progression of Transoral Neck Surgery in Brazil and describe the profile of surgeons involved in this new approach.

## MATERIAL AND METHODS

This is a survey study by descriptive statistics. 

A web based survey was designed to describe the characteristics of surgeons performing TOETVA in Brazil. This was then distributed electronically to a working group of 66 surgeons familiar with TOETVA in Brazil. All surgeons (n=66) were part of a group called “TOETVA-BRA,” communicating via WhatsApp, a common social media app used in Brazil. This group was founded in Brazil to share information, individual experience and knowledge about TOETVA and transoral neck surgery among early adopters. At the time of this writing, it is composed of 127 Brazilian surgeons including several hospital teams and affiliations. The survey was hosted on the REDCap (Research Electronic Data Capture) platform of Santa Casa de Sao Paulo School of Medical Sciences and it was shared by a link, posted in the group. The survey was applied between July and September 2021.

The collected data is decsribed on Table 1.

**Table 1:** Data collected on the REDCap based electronic survey.

TOETVA AND TOEPVA BRAZIL SURVEY		
Name of the surgeon, Birthday, Age	Number of cases which the surgeon felt comfortable with the technique (precisely)	How were surgeons learning if they had no Hands on training
Date of the answers	Number of thyroidectomies among operated cases	Previous observation of cases
City and State of Practice	Number of parathyroidectomies among operated cases	Number of observed cases
Hospital where attended to medical residence	Number of combinated procedures (thyroidectomies + parathyroidectomies)	Participation of a Proctor in the beginning
Medical speciality	Number of central neck dissection among operated cases	Number of cases followed by a Proctor
Date of the first case	Previous training in videosurgery	Percentage of indication of TOETVA among cases of thyroidectomy
Total numbers of cases	Kind of videosurgery training	Percentage of indication of TOEPVA among cases of parathyroidectomy
Number of participation in cases	Experience with robotic surgery	
Participation in cases as a Proctor	Previous practice with videosurgery	Behavior of the surgeon if the patient fits for TOETVA - Shows and explain conventional and endoscopic techniques or not?
Number of cases as a Proctor (approximately)	Previous specific Hands-On training (Specify where)	Final decision between open or endoscopic approach if the patient fits for TOETVA - Surgeon or Patient

All data were compiled and descriptive statistics regarding all topics of the survey were done using REDCap. 

This study has IRB approval from Santa Casa de São Paulo School of Medical Sciences (Number: 5.000.052 | CAAE: 50536121.2.0000.5479) in accordance with the ethical standards of the committee on human experimentation of the Helsinki Declaration of 1975 (revised in 1983). All participants gave written informed consent to participate in this study. 

## RESULTS

The response rate of this survey was 53%, acknowledging that some teams chose to respond to the survey after gathering data from each member. This could result in a lower response rate but one that still reflects a high percentage of eligible participants.

**Figure 2:**
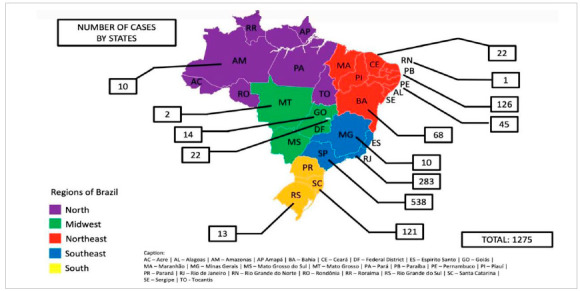
Number of cases by states.

**Figure 3:**
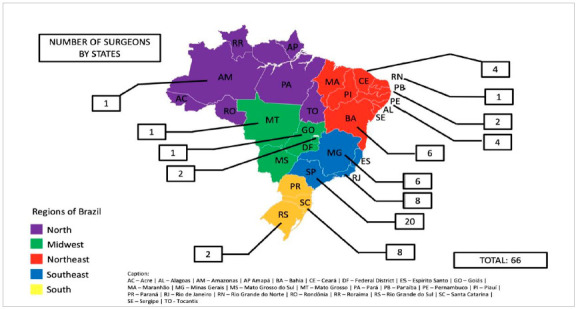
Number of surgeons by states.

According to this survey, to date, 1275 TOETVA/TOEPVA cases had been performed in Brazil. Among the cases, 1229 were thyroidectomies (96.4%), 42 were parathyroidectomies (3.3%) and 4 cases were described as combined procedures (0.3%); central neck dissection was performed in 113 cases (8.8%) (Table 2). Most of the cases were done in the southeast region (821, 64.4%), 538 (42.2%) cases in the State of São Paulo and 283 (22.2%) cases in the State of Rio de Janeiro (Table 2). Surgeons from 14 Brazilian states, including Brasilia (Federal District), and 26 different cities are already performing TOETVA/TOEPVA (Figures 2 and 3).

**Table 2:** Summary of cases performed and surgeon profile.

	Results	
Response rate	53%	
Cases		
Number of cases	1275	
Thyroidectomies	1229 (96.4%)	
Parathyroidectomies	42 (3.3%)	
Combined procedures	4 (0.3%)	
Central neck dissection (Level VI)	113 (8.8%)	
Region	Southeast	821 (64.4%)
	São Paulo	538 (42.2%)
	Rio de Janeiro	283 (22.2%)
Surgeons		
Number of surgeons	66	
Mean age	41	
<50 years	>75%	
Head and Neck	63 (95.5%)	
Oncological	2 (2.9%)	
Endocrine	1 (1.6%)	

Most of the cases are concentrated in the cities of São Paulo, Rio de Janeiro, João Pessoa, and Florianópolis. The number of cases varied from 1 to 203 (median: 6). The largest numbers of cases per group included 203 for one team in São Paulo, 188 for another in Rio de Janeiro, 125 for another one at São Paulo and 126 for a team at João Pessoa. 

Altogether 66 surgeons answered the survey (Table 2). Their age varied from 30 to 63 years old with a median age of 41 years old. More than 75% were under 50 years old, and the predominant age group was between 36 to 47 years old, representing 50% of the sample. According to the survey 95.5% (n=63) are head and neck surgeons, 2.9% (n=2) are oncologic surgeons and 1.6% (n=1) are endocrine surgeons (Table 2). When asked about their participation in real cases of TOETVA/TOEPVA, 30.3% (n=20) participated in up to 5 cases, 37.9% (n=25) in 5 to 25 cases, 13.6% (n=9) in 25 to 50 cases, 4.5% (n=3) in 50 to 75 cases, 6.1% (n=4) in 75-100 cases, 4.5% (n=3) in 100-150 cases and 3% (n=2) had participated in over 150 cases (Table 3). This means that a majority of cases, 86% (n=1101) had been completed by a surgeon who had participated in more than 25 cases. Of note, 30.3% (n=20) of this sample participated as a Proctor in some cases. Trying to understand the individual perception of the learning curve, the surgeons were asked to determine at what case number they became more comfortable with the technique: 28.8% (n=19) said 5 cases, 15.2% (n=10) 10 cases, 15.2% (n=10) 15 cases, 3% (n=2) 20 cases, 1.5% (n=1) 25 cases, 1.5% (n=1) 30 cases, and 34.8% (n=23) said that they are still not comfortable with the technique (Table 3). 

**Table 3:** Results - Surgeons participation in cases and learning curve.

	Results
Surgeons	
Participation in real cases	
Up to 5 cases	20 (30.3%)
5 to 25 cases	25 (37.9%)
25 to 50 cases	9 (13.6%)
50 to 75 cases	3 (4.5%)
75 to 100 cases	3 (4.5%)
100 to 150 cases	3 (4.5%)
> 150 cases	2 (3%)
Participation in cases as Proctor	20 (30.3%)
Individual perception of the learning curve	
5 cases	19 (28.8%)
10 cases	10 (15.2%)
15 cases	10 (15.2%)
20 cases	2 (3%)
25 cases	1 (1.5%)
30 cases	1 (1.5%)
Not comfortable	23 (34.8%)

Altogether 75.8% (n=50) of surgeons said that they had previous videosurgery training before starting TOETVA/TOEPVA (Table 4). Most of them (66%, n=33) had this training during medical residency only, 2% (n=1) did short term courses and 32% (n=16) had both (Table 4). When asked if they were used to perform videosurgery in their daily practice 57.6% (n=38) said yes. Among all surgeons, 27.3% (n=18) were already trained in robotic surgery. The majority of surgeons (80.3%, n=53) involved in this sample have had specific TOETVA training, some in cadaver lab and others in animal lab. Those who didn’t have specific training were already trained in robotic surgery and some went to the USA or Thailand for training and observerships before starting. Most surgeons (85%, n=56) have watched cases from an experienced colleague before performing their first case. The number of observed cases before beginning the technique varied from 1 to 30 (median: 5). The majority (63.6%, n=42) have had a proctor in their first cases and some (15%, n=10) have had a proctor for the first 5 cases (Table 4).

**Table 4:** Results - Surgeons’ training.

	Results
Surgeons	
Video surgery training	
Yes	50 (75.8%)
No	16 (24.2%)
Only during residency	33 (66%)
Short term course only	1 (2%)
Both	16 (32%)
Robotic surgery training	18 (27.3%)
Specific training in TOETVA	53 (80.3%)
Observation of cases	56 (85%)
Participation of a Proctor in the first cases	42 (63.6%)

With regards to surgical indications for TOETVA among Brazilian surgeons, 43.9% (n=29) believe that it is indicated for up to 10% of thyroid cases, 24.2% (n=16) for up to 25%, 16.7% (n=11) for up to 50%, 9.1% (n=6) for up to 75 and 6,1% (n=4) believe that it is indicated for 90% of cases (Table 5). Among parathyroid surgery 75.4% (n=49) believe that it is indicated for up to 10%, 10.8% (n=7) for up to 25%, 7.7% (n=5) for up to 50% and 6,2% (n=4) for up to 75%. When asked about proposing open and endoscopic surgery to a patient who is a candidate for TOETVA/TOPEVA, most surgeons (90%, n=60) said that they expose both ways to the patient, and 95.5% (n=63) let the patient make the choice (Table 5). 

**Table 5:** Results - Indication of TOETVA and TOEPVA.

	Results
Surgeons	
Individual perception of indication for TOETVA	
Indicated for up to 10% of cases	29 (43.9%)
Indicated for up to 25% of cases	16 (24.2%)
Indicated for up to 50% of cases	11 (16.7%)
Indicated for up to 75% of cases	6 (9.1%)
Indicated for up to 90% of cases	4 (6.1%)
Individual perception of indication for TOEPVA	
Indicated for up to 10% of cases	49 (75.4%)
Indicated for up to 25% of cases	7 (10.8%)
Indicated for up to 50% of cases	5 (7.7%)
Indicated for up to 75% of cases	4 (6.2%)
Always expose both techniques to the patient	60 (90%)
Let the patient choose the technique when their case is suitable for the endoscopic approach	63 (95.5%)

## DISCUSSION

Transoral neck surgery for thyroid and parathyroid is a new technique and it is being adopted on a worldwide scale, specially in Asia and the Americas^1^ ^,^ ^2^ ^,^ ^14^ ^,^ ^15^ ^,^ ^26^. In Brazil the first documented cases were done in 2017^6^ but only in 2020 did a larger number of surgeons start to use the technique. The cosmetic aspect of avoiding a cervical scar is an advantage of TOETVA/TOEPVA. Populations who are concerned about cosmesis are more likely to choose a cosmetically superior technique, such as TOETVA. To date, 1275 cases have been completed across all regions of Brazil and most states, consistent with the rapid adoption cited by Banucchi et al.^24^. 

Historically, many surgeons were concerned about a high rate of infection with this technique. To date, the available data do not demonstrate a higher occurrence of infection in TOETVA^27^. Increased surgical time in TOETVA has been observed when compared to conventional surgery. This finding is similar to earlier endoscopic surgeries such as sinus surgery and laparoscopic surgery, which have become standard of care when compared to the open approach^28^. After a short learning curve of 10 to 15 cases^8^ ^,^ ^9^ surgery time decreases and becomes only slightly longer than open thyroidectomy^8^ ^,^ ^9^. Despite these findings, there remains some resistance to the technique that does not appear to be supported by the limited complications to date^29^. 

All surgeons who answered this survey are early adopters of TOETVA^30^. A total of 66 surgeons answered the survey, but that number is probably underestimated, since the survey was only applied to a specific group of surgeons. However the pioneers and those who have a high volume of TOETVA cases in Brazil were included in this sample. Surgeons under the learning curve were also included, which means that numbers will probably grow even faster and this procedure will spread even more rapidly after these surgeons complete their initial learning curve. 

Training background is a critical aspect when starting a new surgical technique. Even with previous videosurgery training, the pioneers of TOETVA in Brazil^6^ ^-^ ^12^ sought training and observerships in other countries. Some went to the USA and others travelled to Thailand, the first high volume centers of TOETVA in the world. These surgeons have collaborated with each other in their first cases and have also developed the first Brazilian courses about TOETVA, using animal lab and cadaver lab which have allowed other surgeons to train without travelling abroad.

The predominant age group between respondent surgeons was 36 to 47 years old, showing that younger surgeons were most likely to adopt TOETVA/TOEPVA. Any new surgical procedure has an initial resistance for various reasons. Most of the adopters have finished their residency at least 5 years prior to initiating TOETVA. Those surgeons learning TOETVA may have had sufficient open cases after these years to advance to remote access techniques, consistent with international recommendations^5^. 

Most surgeons of this sample had some experience in videosurgery training during their residency (75%, n=50) and others underwent short term courses or both. Older surgeons didn’t have videosurgery training during their residency, since it was incorporated during the 2000s. This could explain why older surgeons were less represented in our survey. More than half were used to perform videosurgery in their daily practice before starting TOETVA. Most surgeons observed some cases before starting their own, and the vast majority had proctoring for the earliest cases. 

These aspects may be different in other countries and should vary according to the medical training program. In Brazil, most head and neck surgeons undergo a 2 year general surgery program before applying to head and neck surgery, while a smaller group comes from otolaryngology. Nowadays, general surgery offers some practice of videosurgery due to laparoscopy, and during otolaryngology residency a good number of procedures involve video instrumentation. Regardless of residency, minimally invasive surgery and videosurgery are increasingly common, suggesting that future surgeons will probably learn TOETVA more easily than pioneers and early adopters did.

Regarding applicability of TOETVA, 31.9% of surgeons consider performing TOETVA for, at least, 50% of all thyroid cases, which means that this technique can be offered to a large number of patients. Patient selection is fundamental and experience will certainly bring greater confidence to indicate TOETVA/TOEPVA for a broader but appropriate spectrum of thyroid and parathyroid diseases. 

Among respondents, almost 90% offer both open and transoral procedures when a patient is a candidate for either approach, and 95% of those let the patient decide which one is better. Such an approach honors the individual priorities of each patient, and highlights the promise of individualized decision making. 

Limitations of this study include selection bias of respondent surgeons, since there are likely more surgeons performing TOETVA/TOEPVA that didn’t join TOETVA-BRA WhatsApp group. This group also represents only colleagues of those early adopters of the technique given that they were heavily involved in subsequent training. It is possible that some respondents also overestimated or underestimated the number of cases and other data submitted.

## CONCLUSION

Transoral Thyroid and Parathyroid Neck Surgery are becoming popular in Brazil. To date, 1275 cases have already been performed in all 5 regions of Brazil and in more than half of the federation units. Young surgeons, especially those between 30 and 50 years old, were more likely to adopt these novel approaches. The personal background of videosurgery, specific TOETVA training, observation of an experienced surgeon and proctorship during the first cases seem to be highlights of those who have adopted the technique.

## References

[1] Zhang D, Park D, Sun H, Anuwong A, Tufano R, Kim HY (2019). Indications, benefits and risks of transoral thyroidectomy. Best Pract Res Clin Endocrinol Metab.

[2] Dionigi G, Chai YJ, Tufano RP, Anuwong A, Kim HY (2018). Transoral endoscopic thyroidectomy via a vestibular approach why and how?. Endocrine.

[3] Anuwong A, Sasanakietkul T, Jitpratoom P, Ketwong K, Kim HY, Dionigi G (2018). Transoral endoscopic thyroidectomy vestibular approach (TOETVA) indications, techniques and results. Surg Endosc.

[4] Anuwong A (2015). Transoral Endoscopic Thyroidectomy Vestibular Approach A Series of the First 60 Human Cases. W J Surgery.

[5] Berber E, Bernet V, Fahey TJ III, Kebebew E, Shaha A, Stack BC (2016). American Thyroid Association Statement on Remote-Access Thyroid Surgery. Thyroid.

[6] Tesseroli MAS, Spagnol M, Sanabria Á (2018). Tireoidectomia endoscópica transoral por acesso vestibular (TOETVA): experiência inicial no Brasil. Rev Col Bras Cir.

[7] Lira RB, De Cicco R, Rangel LG, Bertelli AA, Silva GD, Vanderlei JP de M (2021). Transoral endoscopic thyroidectomy vestibular approach Experience from a multicenter national group with 412 patients. Head Neck.

[8] Bertelli AAT, Rangel LG, Lira RB, Tesseroli MAS, Santos IC, Silva GD (2021). Trans Oral Endoscopic Thyroidectomy Vestibular Approach (TOETVA) in Brazil Safety and complications during learning curve. Arch Endocrinol Metab.

[9] Lira RB, Ramos AT, Nogueira RMR, de Carvalho GB, Russell JO, Tufano RP (2020). Transoral thyroidectomy (TOETVA) Complications, surgical time and learning curve. Oral Oncol.

[10] Bertelli AAT, Rangel LG, Araujo GA, Monteiro RC, Massarollo LCB, Russell JO (2019). Transoral endoscopic thyroidectomy by vestibular approach (TOETVA): initial experience in an academic hospital. Arch. Head Neck Surg.

[11] Cicco RD, Souza RP de, Guerra FLB (2020). Transoral endoscopic thyroidectomy vestibular approach: initial experience and comparison with conventional thyroid surgery. Arch. Head Neck Surg.

[12] Gomes MA, Silva GD (2018). Tireoidectomia endoscópica pelo acesso transvestibular (TOETVA). Rev Relato Casos do CBC.

[13] Witzel K, Von Rahden BHA, Kaminski C, Stein HJ (2008). Transoral access for endoscopic thyroid resection. Surg Endosc Other Interv Tech.

[14] Russell JO, Razavi CR, Shaear M, Chen LW, Lee AH, Ranganath R (2019). Transoral Vestibular Thyroidectomy Current State of Affairs and Considerations for the Future. J Clin Endocrinol Metab.

[15] Anuwong A, Ketwong K, Jitpratoom P, Sasanakietkul T, Duh Q-Y (2018). Safety and Outcomes of the Transoral Endoscopic Thyroidectomy Vestibular Approach. JAMA Surgery.

[16] Jitpratoom P, Ketwong K, Sasanakietkul T, Anuwong A (2016). Transoral endoscopic thyroidectomy vestibular approach (TOETVA) for Graves' disease a comparison of surgical results with open thyroidectomy. Gland Surg.

[17] Rattner D, Kalloo A, ASGE/SAGES Working Group (2006). ASGE/SAGES working group on natural orifice translumenal endoscopic surgery. Surg Endosc.

[18] Richmon JD, Pattani KM, Benhidjeb T, Tufano RP (2011). Transoral robotic-assisted thyroidectomy A preclinical feasibility study in 2 cadavers. Head Neck.

[19] Richmon JD, Holsinger FC, Kandil E, Moore MW, Garcia JA, Tufano RP (2011). Transoral robotic-assisted thyroidectomy with central neck dissection Preclinical cadaver feasibility study and proposed surgical technique. J Robot Surg.

[20] Banuchi VE, Ballakur SS, Russell JO, Tufano RP (2020). Benefits and risks of scarless thyroid surgery. Ann Thryoid.

[21] Russell JO, Clark J, Noureldine SI, Anuwong A, Al Khadem MG, Yub Kim H (2017). Transoral thyroidectomy and parathyroidectomy - A North American series of robotic and endoscopic transoral approaches to the central neck. Oral Oncol.

[22] Juarez MC, Ishii L, Nellis JC, Bater K, Huynh PP, Fung N (2019). Objectively measuring social attention of thyroid neck scars and transoral surgery using eye tracking. Laryngoscope.

[23] Chen LW, Razavi CR, Hong H, Fondong A, Ranganath R, Khatri S (2020). Cosmetic outcomes following transoral versus transcervical thyroidectomy. Head Neck.

[24] Banuchi V, Vlaming K-A (2020). Transoral endoscopic thyroidectomy-vestibular approach starting a program in an inner-city hospital. Ann Thyroid.

[25] Cuello NL, Gentile SC, Fernández Ranvier G, Servicio de Cirugía Endocrina y Metabólica (2019). Mount Sinai Hospital New York. USA, Servicio de Cirugía de Cabeza y Cuello. Clínica Provincial de Merlo. Buenos Aires. Argentina. Primera tiroidectomía transoral vestibular endoscópica (TOETVA) en la Argentina. Rev Argent Cir.

[26] Russell JO, Razavi CR, Garstka ME, Chen LW, Vasiliou E, Kang S-W (2019). Remote-Access Thyroidectomy A Multi-Institutional North American Experience with Transaxillary, Robotic Facelift, and Transoral Endoscopic Vestibular Approaches. J Am Coll Surg.

[27] Karakas E, Klein G, Michlmayr L, Schardey M, Schopf S (2022). Transoral thyroid surgery vestibular approach is there an increased risk of surgical site infections?. Updates Surg.

[28] Bertelli AAT, Lira RB, Gonçalves AJ, Russell JO, Tufano RP, Dionigi G (2021). Transoral endoscopic thyroidectomy vestibular approach (TOETVA) pioneers's point of view. Arch Endocrinol Metab,.

[29] Tincani A, Lehn C, Cernea C, Queiroz E, Dias F, Walder F (2021). Transoral thyroidectomy A reflexive opinion on the technique. Arch Endocrinol Metab.

[30] McCulloch P, Altman DG, Campbell WB, Flum DR, Glasziou P, Marshall JC (2009). No surgical innovation without evaluation the IDEAL recommendations. Lancet.

